# Real world outcomes of photodynamic therapy for chronic central serous chorioretinopathy

**DOI:** 10.1038/s41433-022-02370-2

**Published:** 2022-12-26

**Authors:** Samir Khandhadia, Suresh Thulasidharan, Nguyen Thuy Vy Hoang, Sameh Alkhalili Ibrahim, Yanling Ouyang, Andrew Lotery

**Affiliations:** 1 University Hospital Southampton NHS Foundation Trust, Southampton, UK; 2 Faculty of Medicine, University of Southampton, Southampton, UK

**Keywords:** Outcomes research, Drug therapy, Retinal diseases, Health services, Vision disorders

## Abstract

**Objectives:**

We describe the real-world outcomes of photodynamic therapy (PDT) for chronic central serous chorioretinopathy (CSCR) in a single centre over nine years.

**Methods:**

We carried out a retrospective analysis of patients with chronic CSCR who received half dose PDT in a single centre between 2011 and 2019. Visual acuity (VA) and retinal thickness (RT) was recorded between baseline visit and first recorded review visit.

**Results:**

We included 125 eyes of 113 patients in this study. Mean age at treatment was 55.0 ± 12.1 years, with a higher male predominance (83 men, 30 women). Mean baseline VA was 0.40 ± 0.31 logMAR with a mean visual outcome gain post-PDT of 0.05 logMAR (*p* = 0.005). Mean baseline RT was 390 ± 82 microns with a mean reduction of RT post-PDT of 66 microns (*p* < 0.001). 17.6% of eyes were treated for recurrent CSCR.

**Conclusion:**

We found overall a mean improvement in VA and structural outcomes after PDT. In the absence of randomised clinical trials this study supports the use of half dose PDT for treatment of chronic CSCR.

## Introduction

Central serous chorioretinopathy (CSCR) is an enigmatic idiopathic condition characterised by remitting and relapsing accumulation of subretinal fluid, leading to a serous neurosensory detachment. If fovea-involving this can lead to visual symptoms, such as decreased vision, distortion, scotoma and reduced colour vision. CSCR often resolves spontaneously, however may persist. Chronic CSCR is defined as persistent serous detachment of duration 4 to 6 months.

The pathophysiology of CSCR has not been fully elucidated, but key features include choroidal hyperpermeability and engorgement, focal defects in the retinal pigment epithelium (RPE) and the accumulation of subretinal fluid from the choroid [1]. Photodynamic therapy with verteporfin (PDT) is widely utilised as a therapy despite the lack of robust randomised clinical trials to confirm efficacy.

PDT induces free radical production from laser-induced activation of a photoactive chemical, Verteporfin (Visudyne^®^). Verteporfin is benzoporphyrin derivative monoacid which absorbs the light energy to generate a short-lived singlet oxygen as free radicals that leads to local vascular occlusion and cell damage. Following uptake, verteporfin is selectively retained in the rapidly proliferating endothelium of choroidal neovascularisation and with localized light activation leads to closure of the mature neovascular proliferation. PDT, thus, was initially developed for ocular use to treat neovascular age-related macular degeneration (nAMD) [2], however this indication has been superseded by anti-vascular endothelial growth factor (anti-VEGF) therapies for nAMD. Half dose PDT has been used successfully (off-licence) for treatment of CSCR and is currently the treatment of choice for long-term resolution [3–9].

PDT for treatment of CSCR is a two-step process. Initially the photoactive chemical, Verteporfin is administered via an intravenous infusion. As verteporfin courses through the choroidal vasculature, a specific wavelength laser (689 nm) is applied to areas of leakage as identified by fundus fluorescein angiogram (FFA) and / or indocyanine green angiogram (ICGA). To reduce risk of long-term foveal atrophy, half dose / fluence / time PDT can be used, with half dose being the preferred option [10]. The free radical production within the choroidal vasculature leads to localised damage and remodelling of the choroidal vasculature. This in turn reduces choroidal perfusion and subsequently reduces fluid leakage under damaged retinal pigment epithelial cells [10, 11].

There are limited large scale studies of the use of PDT in treating CSCR. Therefore, in this study we report the real-world results with half dose PDT in chronic CSCR in a large cohort of patients.

## Methods

Patients were identified from a retrospective search of electronic medical records at Southampton Eye Unit, Southampton, UK. We included patients who received photodynamic therapy for CSCR between January 2011 to November 2019 (107 months). We excluded any eyes which had received anti-VEGF injections either before or after PDT, and also any patient receiving non-protocol PDT (i.e. full dose or modified fluence), to standardize this patient cohort.

Fluorescein and indocyanine angiograms were performed to localise and define the point of active leakage. Each patient was given half-dose PDT based on a standard protocol. First an intravenous infusion of verteporfin was given over 10 min, at half the standard dose calculated as 3 mg/m^2^ of body surface area (BSA). The BSA is based on the patient body weight and height and was calculated using a standard BSA nomogram or Visudyne BSA Slide rule calculator. Five minutes after the end of the infusion, a non-thermal laser (Quantel Medical Activis) of 689 nm wavelength was applied via a contact lens (Area Centralis) on a slit-lamp based delivery system, over the area of leakage based on the angiogram findings. The treatment spot size was determined by the greatest linear dimension (GLD) of the leakage in the earlier images of the angiogram, with a surrounding margin of 500 microns. A safety margin of 200 microns from the temporal edge of optic disc to nasal margin of the treatment spot was maintained to avoid damage to the optic disc. A magnification factor was also applied based on the contact lens used. Standard fluence setting was used (50 J/m2), as was standard time (83 s). Patients were instructed to avoid exposure to direct sunlight, wear sunglasses, and cover their skin for 48 h following treatment, to avoid photosensitive reactions.

The primary outcome measure was change in visual acuity (VA) at first recorded follow-up post-PDT, compared to baseline. Secondary outcome measures included change in retinal thickness (RT), and any complications. RT was calculated as the subfield of maximal leakage on the FFA. The same subfield was used for pre and post-PDT measurements. Heidelberg Spectralis and Topcon 3D OCT scans were used. To enable comparison between the two machines, 50 microns was added onto Topcon measurements [12]. Subfoveal choroidal thickness was measured from basement membrane to sclera using callipers. Retinal thinning was defined as subfields on the macular map showing retinal thickness below normative data.

We excluded any eyes which had received anti-VEGF injections either before or after PDT to standardise the patient population. SPSS (V28, IBM Ltd) was used for statistical analysis. Statistical significance was taken as *p* < 0.05. Mean VA / RT pre and post PDT were compared using the 2-sided t-test. Correlation was carried out between continuous variables using the Pearson correlation coefficient. Linear regression (stepwise method) was carried out using continuous dependent variables.

## Results

**Fig. 1:**
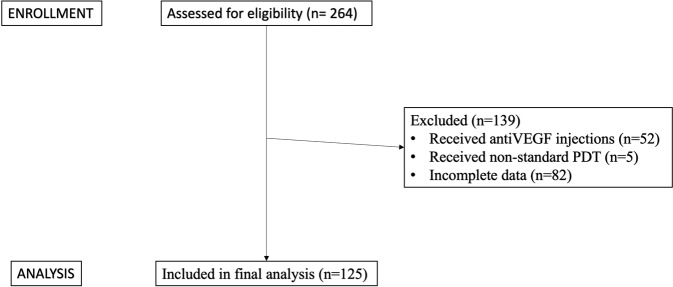
Consort diagram summarising selection of eligible patients.

Out of 264 eyes initially identified from electronic medical records who received PDT for CSCR, we included 125 eyes of 113 patients. We excluded 52 eyes which had received anti-VEGF injections (either before or after PDT), 5 eyes which received non-standard PDT (full dose / modified fluence), and 82 eyes with incomplete data. (Consort diagram, Fig. 1)

Mean age at treatment was 55.0 + /− 12.1 years, with higher male predominance (83 men, 30 women). Average time to review following PDT was 24 + /− 13 weeks (range 2–87 weeks). 12 patients (9.6%) had bilateral PDT on the same sitting (this was done sequentially in one sitting, the most symptomatic eye treated first).

**Fig. 2:**
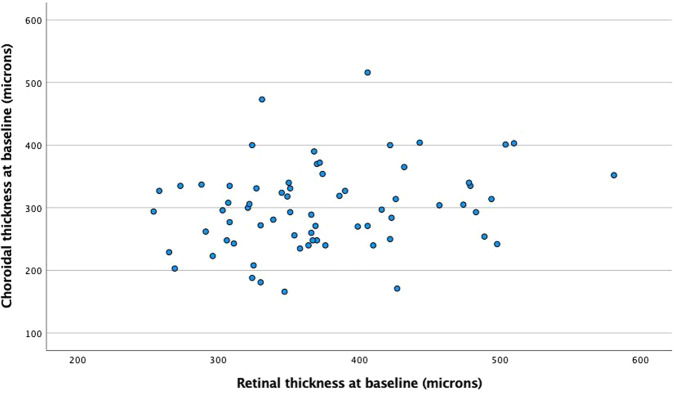
Baseline retinal thickness vs baseline choroidal thickness.

All patients underwent FFA, a minority (19%, *n* = 24) also underwent ICGA. ICGA was not used if there was a potential allergy to indocyanine green and/or physician preference to just perform FFA. Most eyes had fovea involving CSCR (74%, *n* = 93) and exhibited a single area of leakage on FFA (92.8%, *n* = 116). GLD of the leaking area ranged between 200 to 4600 microns (mean 2148 + /− 1240 microns). The OCT morphology is shown in Table 1. Baseline choroidal thickness was available for 68 eyes (only available on Heidelberg OCT with enhanced depth imaging). Choroidal thickness for these eyes was 300 + /− 67 microns. Baseline choroidal thickness was not correlated with baseline RT (*r* = 0.238, *p* = 0.051, Fig. 2).

**Table 1:** OCT morphology at baseline.

OCT MORPHOLOGY	Number	%
Subretinal fluid (SRF) alone	98	78.4
SRF + hyperreflective deposit	6	4.8
SRF + intraretinal fluid (IRF)	5	4
SRF + IRF + hyperreflective deposit	1	0.8
SRF + IRF + pigment epithelial detachment (PED)	1	0.8
SRF + PED	6	4.8
SRF + epiretinal membrane	1	0.8
SRF + PED + hyperreflective deposit	2	1.6
Other: Schisis	1	0.8
Unknown (OCT scans lost)	4	3.2

**Fig. 3:**
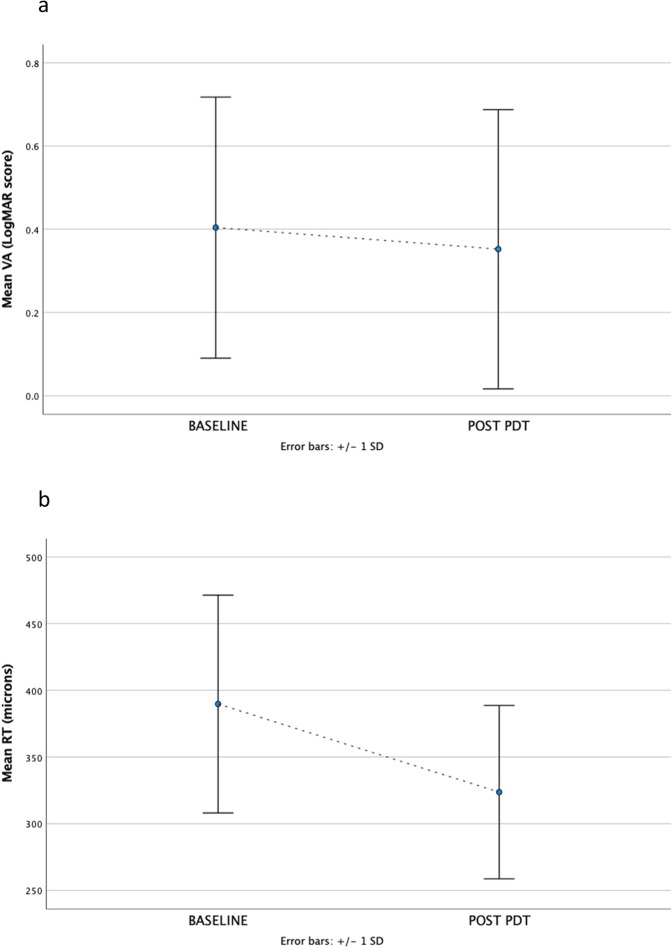
Functional and structural changes from baseline to post PDT laser. Panel **a** shows change in mean visual acuity (VA) from baseline to post PDT laser. Panel **b** shows change in mean retinal thickness (RT) from baseline to post PDT laser.

**Fig. 4:**
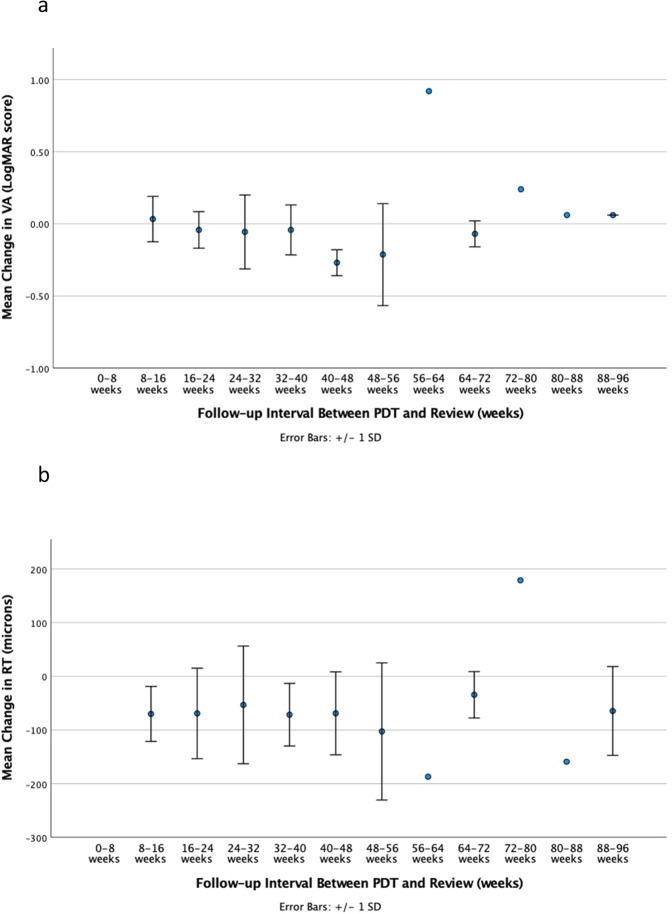
Functional and structural changes based on follow-up interval for whole cohort. Panel **a** shows change in mean visual acuity (VA) between PDT laser and review. Panel **b** shows change in mean retinal thickness (RT) between PDT laser and review.

There was a mean improvement in VA of 0.05 logMAR following PDT (mean baseline VA 0.40 + /− 0.31 logMAR vs mean post-PDT VA 0.35 + /− 0.33 logMAR, *p* = 0.005, Fig. 3a). Of these, 9.6% eyes (*n* = 12) exhibited a gain in vision of 3 lines (0.3 logMAR) or more, however 4.8% (*n* = 6) eyes had a reduction in vision of 3 lines (0.3 logMAR) or more. The review time from PDT for the patients who lost 3 lines or more logMAR vision, was similar to the whole cohort at 34 + /− 21 weeks. There was also a reduction in mean RT following PDT of 66 microns (mean baseline RT 390 + /− 82 microns vs mean post-PDT RT 324 + /− 65 microns, *p* < 0.001, Fig. 3b). 22 eyes (17.6%) were treated for recurrent CSCR, 7 of these on the 2nd recurrence, and 2 on the 3rd recurrence. VA and RT outcomes in the whole cohort was not substantially different based on follow-up interval (Change in VA vs follow-up interval: *r* = −0.006, *p* = 0.943, Fig. 4a. Change in RT vs follow-up interval: *r* = 0.019, *p* = 0.830, Fig. 4b).

Linear regression was carried out using either change in VA or change in RT (from pre- to post-PDT), as the dependent variable. Independent variables included age, gender, baseline VA, baseline RT, foveal involvement, baseline choroidal thickness, and GLD. Change in VA could not be predicted by any of these independent variables. However, change in RT could be predicted by reduced baseline RT (*B* = −0.901, 95% CI −1.155 to −0.647, *p* < 0.001).

Localised retinal thinning was noted on the first follow-up in 26 eyes (20.8%), and generalised thinning in 7 eyes (5.6%). There were no complications of the PDT procedure itself, either from verteporphin or laser, in particular there were no anaphylactic reactions.

## Discussion

Our retrospective review of 125 eyes of 113 patients with chronic CSCR showed benefit of half-dose PDT in improving both VA and RT.

This compares favourably to other published studies. Van Dijk et al. reported a prospective randomised controlled clinical trial (the PLACE study) in which 89 eyes of 89 patients with chronic CSCR demonstrated a + 7 ETDRS letter improvement 7–8 months post-half dose PDT, with 67% of eyes showing complete resolution of subretinal fluid [3].

Several retrospective case series report similar findings. Lai et al. (2015) reported in a retrospective case series, a mean VA improvement from 0.35 to 0.14 logMAR, in 75 eyes after 3 years of half dose PDT, with 93% eyes exhibiting complete resolution of SRF at the final visit [8]. Lai et al. (2016) in a retrospective case series reported 136 eyes of 123 chronic CSCR patients treated with half dose PDT exhibited an improvement in VA from 0.36 to 0.15 logMAR, 36 months following treatment from half dose PDT, 97% of eyes with complete resolution of SRF at this point [8]. Fujita et al (2015) reported a retrospective case series of 204 eyes of 204 chronic CSCR patients treated with half dose PDT, with a mean improvement of VA from 0.11 to −0.01 at 12 months (*p* < 0.0001), with 89% with complete resolution of SRF at final follow-up [7].

Although we found no predictors for VA change, we did find that lower baseline RT predicted RT change. This suggests CSCR with less leakage may respond better to PDT. Retinal thinning was demonstrated in 20.6% eyes post-PDT, It is likely this was present pre-PDT, however was not easily measurable from the thickness map due to the presence of pre-treatment subretinal fluid. Outer retinal thinning is often seen in CSCR and is a predominant cause of long-term visual loss.

Drawbacks of this study include the following. Both Topcon 3D-OCT and Heidelberg Spectralis OCT machines were used for assessing macular thickness, based on the technological evolution and access to the equipment during the nine years period of this study. Although a conversion factor was used (Heidelberg OCT thickness calculated as 50 microns more than Topcon OCT thickness as mentioned in Methods above), this may still impact on RT outcomes, especially if different machines were used pre and post PDT.

Variability in operator techniques and different angiogram machines used to calculate the greatest linear dimension may also introduce some bias, although all operators performed PDT under a standard protocol.

We used half dose PDT rather than half fluence PDT as this delivered the same overall energy to the retina but used less verteporfin. This therefore reduced the risk of drug related complications such as photosensitivity reactions.

Several patients were referred to us from out of area eye departments. Such patients were followed for a longer time locally before being referred for PDT. Longer duration of CSCR may lead to worse outcomes, as reported by Li et al. [13]. This may explain the lack of significant improvement in visual acuity in our study population.

The standard planned time to review post PDT was 3 months. However, patients who were initially referred from out-of-are eye centres were usually followed up locally. This resulted in the large variation in time to review observed. Nevertheless, we did not find VA and RT outcomes were substantially affected by follow-up interval.

More long-term prospective randomised placebo controlled clinical trials would show the long-term benefits / risks of half-dose PDT, particularly in terms of the reactivation rate / extent of foveal atrophy as a complication of treatment. Currently there is a lack of robust placebo controlled RCTs to guide clinicians on what the most effective therapy is for CSCR. Non-placebo-controlled trials such as this suggest that half dose PDT is effective in CSCR. However, many departments do not have access to photodynamic therapy, indeed at the time of writing there is an international shortage of verteporfin [14].

This study adds to the current literature with a series of patients who have had half dose PDT to treat CSCR. The results are encouraging that this treatment stops further deterioration and can improve visual acuity and structural outcomes. The improvement in visual acuity was modest although some patients had a significant 3-line gain in vision. However, a limitation is the lack of a placebo control group. As CSCR is a relapsing condition, some of the improvement noted may simply reflect the natural history of the disease. Nevertheless, these results are sufficiently encouraging to suggest that placebo controlled clinical trials of half dose PDT laser should be undertaken in CSCR. Such studies would encourage greater adoption of this therapy.

Alternative therapies are very limited. High density subthreshold micropulse (HDSM) laser can target focal areas of RPE leak without damaging the overlying photoreceptors and has been suggested as an option. However, several studies have reported reduced benefit outcomes of HDSM compared to half-dose PDT in the treatment of chronic CSCR [3, 15]. Thermal laser photocoagulation has also been used in CSCR with an extrafoveal leak but carries with it the risk of choroidal neovascularisation. Eplerenone, a mineralocorticoid antagonist, has been recommended as a potential treatment option for CSCR, however recent randomised clinical trials have showed no benefit of oral Eplerenone either vs placebo [16] or PDT [17].

In conclusion, this retrospective case series reports the effect of half dose photodynamic therapy in chronic CSCR. The results described here support further investigation of this treatment in CSCR, particularly in the absence of alternative therapies.

## Summary

### What was known before

- Photodynamic therapy with verteporfin (PDT) is currently utilised as off-label treatment for chronic central serous retinopathy (CSCR) despite the lack of robust randomised clinical trials to confirm efficacy.

### What this study adds

- Our real-world retrospective review of 125 eyes of 113 patients with chronic CSCR shows benefit of half-dose PDT in improving both VA and CRT.

## Data Availability

The datasets generated during and/or analysed during the current study are available from the corresponding author on reasonable request.
